# E6/E7 mRNA testing for human papilloma virus-induced high-grade cervical intraepithelial disease (CIN2/CIN3): a promising perspective

**DOI:** 10.3332/ecancer.2015.533

**Published:** 2015-04-29

**Authors:** Massimo Origoni, Paolo Cristoforoni, Guia Carminati, Chiara Stefani, Silvano Costa, Maria Teresa Sandri, Luciano Mariani, Mario Preti

**Affiliations:** 1 Department of Gynaecology & Obstetrics, Vita Salute San Raffaele University, School of Medicine, Milano 20132, Italy; 2 Polo Oncologico Villa Montallegro, Genova 16145, Italy; 3 M.F. Toniolo Hospital, Bologna 40141, Italy; 4 Division of Laboratory Medicine, European Institute of Oncology, Milano 20141, Italy; 5 HPV-UNIT, Regina Elena National Cancer Institute, Roma 00144, Italy; 6 Unit of Preventive Gynaecology, European Institute of Oncology, Milano 20141, Italy

**Keywords:** human papillomavirus, HPV, E6, E7, mRNA, cervical cancer, HPV-DNA

## Abstract

Since the introduction of biomolecular testing for the identification of high-risk human papillomavirus DNA (hrHPV-DNA) in cervical cancer preventive strategies, many interesting aspects have emerged in this field; firstly, HPV-DNA testing has been demonstrated to have better sensitivity than conventional cytology in several settings: screening, triage of ASC-US and in follow-up after treatment. Despite this, some limitations of these new technologies have also been underlined: the major issue is the low specificity of the tests, which cannot discriminate between regressive and progressive infections. Thus, recent research has moved the attention towards novel markers of progression that could more precisely detect cases at real risk of cancer development. In view of the fact that progression to cancer is dependable of the E6/E7 proteins integration and transforming action, the overexpression of E6/E7 transcripts has been seen as a valuable marker of this risk. This review aims to summarise the literature data on this topic and to provide a clear view of the emerging perspectives.

## Background

To reduce morbidity and mortality caused by cervical cancer, in the past 40 years, most developed countries have introduced cervical screening programmes based on cytological examination of cervical smears (Pap test). However, cervical cytology demonstrated a less than optimal sensitivity, and advances in the knowledge of the natural history of the disease have more recently suggested alternative approaches. Persistent infection with high-risk genotypes of the human papillomavirus (hrHPV) has been recognised as the necessary cause of cervical cancer [1]. This led initially to the adoption of molecular tests evaluating hrHPV-DNA presence for the triage of minimally abnormal Pap smears. More recently, evidence has accumulated which implies that a significant improvement of the effectiveness of cervical cancer screening can be achieved by using hrHPV-DNA testing as a primary screening tool. As a matter of fact, hrHPV-DNA testing has a better sensitivity for clinically relevant cervical lesions (cervical intraepithelial neoplasia 2 or worse, CIN2+) than cytology. Moreover, molecular testing proved to offer better protection against high-grade CIN and cancer than cytology in subsequent screening rounds, allowing extension of the screening intervals with favourable logistic and economic implications. Nonetheless, hrHPV infections are rather common in the general population and most of them are transient, spontaneously regressing in a 18–24 months’ period. In fact, the positive predictive value (PPV) of a positive hrHPV-DNA test is rather low (i.e., only a small proportion of the women who test positive for high-risk HPV-DNA will have CIN2+ lesions at the time of the test or will develop it in the next few years), and up to 10% of the screened population test positive and will consequently need some form of second-level triage. The research effort has therefore been focused on potentially alternative or supplementary testing methods capable to limit unnecessary follow-up procedures of women with clinically not relevant, transient hrHPV infections. So far, most of the national guidelines on cervical cancer prevention consider reflex cytology (i.e., the lecture of the cytological sample collected at the time of primary HPV-DNA testing but stored and not initially evaluated) a valuable triage tool for hrHPV-DNA-positive women. Nevertheless, up to 8% of hrHPV-positive women with normal cytology have or will develop in the subsequent years a CIN2+ lesion, and the previous knowledge of HPV-DNA status might influence the subjective reading of cytology. Therefore, there is a strong need for biomarkers that will allow risk-stratification of HPV-positive women with normal cytology or, alternatively, capable of replacing cytology as a triage tool. Considering that the progression to cervical malignancy requires the overexpression of the E6 and E7 genes of the integrated hrHPV genome, demonstration in cervical samples of hrHPV E6/E7 transcripts might be more specific than hrHPV-DNA testing alone for the detection of CIN2+ lesions. Nowadays, transcript analysis is feasible on cervical scrapings, because the introduction of liquid-based cytology has resulted in collection media that preserve RNA sufficiently to allow *in vitro* amplification and detection.

## hrHPV E6/E7 mRNA detection

**Figure 1.**
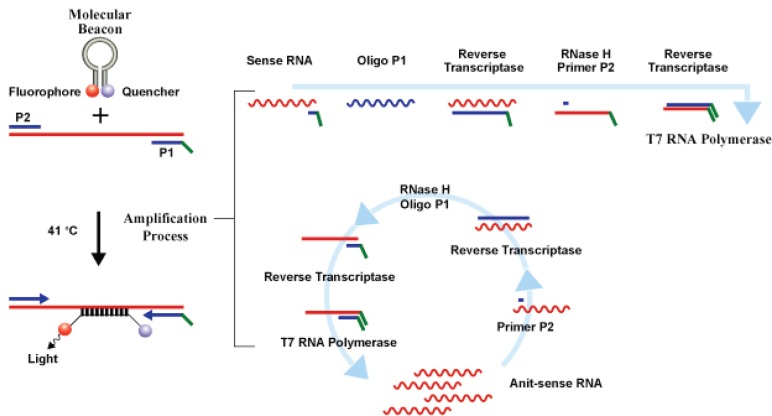
The NASBA RNA amplification technology.

**Figure 2.**
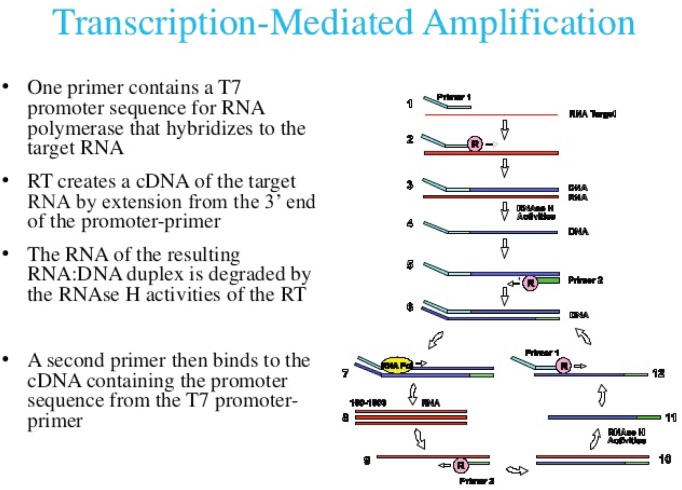
The TMA RNA amplification technology.

Currently, three commercial tests exist for detecting hrHPV E6/E7 messenger RNA (mRNA). The PreTect HPV-Proofer (NorChip AS, Klokkarstua, Norway) and the NucliSENS Easy Q HPV (bioMérieux) are based on the same technology, but are produced by different companies, with small differences in mRNA extraction protocol and data analysis [2]. These tests are nucleic acid sequence-based amplification (NASBA). The assay is an isothermal, RNA real-time amplification method that detects E6/E7 mRNA and perform genotyping of the five more oncogenic hrHPV types (16, 18, 31, 33, and 45) [3]. The NASBA amplification is based on primer extension and transcription by coordinated activities of three enzymes (RNase H, reverse transcriptase and RNA polymerase). The amplification reaction is isothermal and performs at 41°C; due to this relatively low temperature, contaminating DNA cannot be amplified. Real-time detection using molecular beacons is based on the measurement of the time-related detection of increase in a fluorescent signal. This signal is produced only after specific binding of the molecular beacon to an amplicon (specific HPV mRNA) (figure 1). The measurement is performed in combination with the amplification step (real time) [4]. Both tests received the CE-IVD mark. (figure 1) The APTIMA HPV Assay (Hologic, San Diego, CA ) is a target amplification nucleic acid probe test for the qualitative detection of E6/E7 viral mRNA from 14 hrHPV types (16/18/31/33/35/39/45/51/52/56/58/59/66/68). The assay involves three main events in a single tube: target capture, target amplification by transcription-mediated amplification (TMA) and detection of the amplification products. TMA is a transcription-based nucleic acid amplification method that involves two enzymes, reverse transcriptase and RNA polymerase (figure 2). The reverse transcriptase is used to generate a DNA copy of the target mRNA sequence containing a promoter sequence for RNA polymerase. RNA polymerase produces multiple copies of RNA amplicon from the DNA copy template [5]. The detection of the amplicons is performed by the hybridisation protection assay (HPA) using single-stranded nucleic acid probes with chemiluminescent labels that are specifically complementary to the target amplicons. The selection reagent differentiates between hybridised and unhybridised probes by inactivating the label on the unhybridised probes. During the detection step, light emitted from the labelled RNA/DNA hybrids is measured as photon signals in a luminometre and are reported as relative light units (RLU) [6]. APTIMA assay could also perform genotyping for HPV 16 single and 18-45 in pool but requires a separate APTIMA 16, 18/45 genotype assay. The APTIMA HPV assay is a FDA and CE-IVD approved test.

## State of the art

In 2009, a group of researchers from Catholic University, Rome, published their findings on 180 colposcopically and histologically evaluated women, studied for the presence of HPV-DNA (HC II) and mRNA (NucliSENSE) [7]. As expected, fewer women tested positive for mRNA transcripts than hrHPV-DNA, and there was a fairly good correlation between the degree of positivity and the severity of the lesion. DNA from hrHPVs was found in 57.8% of the specimens; E6 and E7 transcripts were found in 45%. The rates of detection of HPV-DNA and of E6 and E7 transcripts were 33.3% and 25%, respectively, for specimens with normal findings; 51.4% and 31.9%, respectively, for specimens with cervical intraepithelial neoplasia grade 1 (CIN1); and 61.1% and 44.2% for specimens with CIN2, respectively. All specimens with CIN3 and 95.5% of specimens from patients with squamous cell carcinoma were positive by both assays. Overall, the mRNA tests showed a better specificity than the DNA tests for high-grade lesions (72.7% and 56.2%, respectively) and a higher positive predictive value (59.3% and 49.0%, respectively). The authors suggested that mRNA assays could be more powerful than DNA testing for predicting the risk of progression and offer a strong potential as a tool for triage and patient follow-up. In 2011, Burger from Norway performed a systematic review to determine the test performance of HPV mRNA testing compared to DNA testing using CIN2+ as the target condition [2]. The reference standard used to diagnose precancerous lesions was histologically confirmed cervical intraepithelial neoplasia 2+ (CIN2+). Sensitivity, specificity, positive and negative likelihood ratios and diagnostic odds ratios were evaluated for each study. The included studies (11 of 3271 publications) were of varying methodological quality and predominately performed in a secondary screening setting. Eight studies investigated the performance of the PreTect HPV-Proofer/NucliSENS EasyQ, two studies investigated the performance of the APTIMA assay, and one study investigated both mRNA tests on the same patient samples (Table 1). Due to few studies and considerable clinical heterogeneity, pooling of data was not possible; the authors, however, compiled a ‘best evidence synthesis’ for E6/E7 mRNA HPV testing. Sensitivities ranged from 0.41 to 0.86 and from 0.90 to 0.95 for the PreTect HPV-Proofer/Easy Q and APTIMA assay, respectively. Specificities ranged from 0.63 to 0.97 and from 0.42 to 0.61 for the PreTect HPV-Proofer/Easy Q and APTIMA assay, respectively. The SROC curves for both mRNA tests were to the left of the diagonal, and the APTIMA assay performed closest to the DNA tests. The authors concluded that mRNA tests have diagnostic relevance, but additional studies and economic evaluations have to be performed in order to make a solid conclusion regarding the clinical applicability of HPV mRNA testing. In 2012, Monsonego published the results from the FASE Study (French APTIMA screening evaluation), a multicentre trial involving 5,000 French women designed to assess the performance of APTIMA HPV assay (AHPV), hybrid capture 2 (HC2), in-house PCR genotyping, and ThinPrep LBC in population-based screening [8]. AHPV had the highest absolute risk of both histological endpoints, detecting 5% to 15% more CIN3+ and CIN2+ lesions, respectively, than LBC. Compared to the HC2 assay, the relative risk of AHPV positivity was 24% to 29% higher, with a significant difference in CIN2+ detection. With LBC as reference, AHPV had the best sensitivity/specificity balance measured by AUC (area under ROC curve) comparison test (significant for CIN2+), and the colposcopy referral rate (9.2%) comparable to that of LBC (8.7%). Data from the FASE study corroborate the suitability of AHPV in a primary cervical cancer screening. Several studies have investigated the PreTect HPV-Proofer assay on biopsy and cervical scraping samples and showed that the ratio of hrHPV E6/E7 mRNA positivity to hrHPV-DNA positivity increased along with the histological severity of dysplasia. This suggests a higher specificity of this mRNA assay for high-grade cervical lesions compared to HPV-DNA assays [9]. In a recent study by Rijkaart [10], 375 women among 13.401 women participating in a population-based cervical cancer screening programme in the Netherlands were stratified for CIN2+ risk according their hrHPV E6/E7 mRNA status. Study populations included 202 women with normal cytology, 88 with borderline or mild dyskaryosis (BMD) and 85 with moderate dyskaryosis or worse (>BMD); all of the women tested positive for hrHPV-DNA by the GP5+/6+ PCR assay used in the screening programme. A positive mRNA test result conferred an increased CIN2+ risk in hrHPV-DNA-positive women with normal cytology (0.55, 95% CI: 0.34–0.76) versus 0.20 (95% CI: 0.07–0.33) in mRNA-negative women. In hrHPV-DNA-positive women with BMD or >BMD, the result of the mRNA test did not influence the CIN2+ risk. Therefore, the authors concluded that mRNA testing by PreTect HPV-Proofer might be of value to select hrHPVDNA- positive women with normal cytology at an increased risk and therefore requiring immediate referral to colposcopy. A very recent study from Korea evaluated in a series of 337 ThinPrep samples a commercial diagnostic kit targeting a HPV E6/E7 mRNA based on RTqPCR assay and reported 91% sensitivity and 98.6% specificity for CIN2+ cervical lesions [11]. The overall positive rates of the HPV E6/ E7 mRNA RT-qPCR assay were 100% (24/24), 86% (38/44), 100% (7/7), 37% (10/27), 15% (5/32), and 1% (3/203) in SCC, HSIL, ASC-H, LSIL, ASC-US, and normal samples, respectively. The tested kit showed a remarkably higher sensitivity (91 vs. 41%) compared to the realtime NASBA assay, considered in the study as the reference standard. The tested assay showed also a better correlation between rate of positivity and severity of the cytological lesion and stressed the possible relevance of mRNA detection assays in the clinical management of women screened for cervical cancer precursors. In the discussion section, however, the authors underlined that the poor performance of the NASBA assay could be related to a series of technical limitations due to the adopted method of RNA extraction and amplification and stressed the importance of larger studies evaluating a more representative patient population as well as the importance of taking into account geographical differences, due to variable HPV genotypes prevalence in cervical cancer specimens. In summary, the performance in terms of sensitivity/specificity of the 5 hrHPV mRNA tests (PreTect HPV-Proofer and NucliSENS EasyQ HPV) cannot be pooled with the 14-h HPV mRNA test APTIMA. The APTIMA test is more similar to HPV DNA test like Hybrid Capture 2 regarding sensitivity and specificity, while the five genotype HPV E6/E7 mRNA tests have much higher specificity than the APTIMA assay [12, 13].

## HPV mRNA testing in the triage of LSIL

HPV-DNA tests have low specificity and a high positivity rate in LSILs, making HPV-DNA test not particularly useful in triage of LSIL. The 14 genotype APTIMA test and especially the five genotype HPV E6/E7 mRNA tests have higher specificity and a low positivity rate in LSIL. A low positivity rate translates into a low referral rate for colposcopy, which is very appealing for triage situations [13–16]. Women with minor cervical lesions have a small but significantly increased risk of developing cervical cancer compared to women with normal smears. The purpose of triage is to identify women needing further follow-up by colposcopy and biopsy to detect high grade cervical dysplasia or cancer (CIN2+). In cases of atypical squamous cell of undetermined significance (ASC-US), it is now widely recognised that triage with an HPV-DNA test is more sensitive, but less specific than repeat cytology [17, 18]. For low-grade squamous intraepithelial lesions (LSIL), however, recommendations regarding the best triage method are conflicting and vary from repeat cytology, HPV testing or direct referral to colposcopy [19, 20]. Meta-analyses indicate that triage of LSIL with a high-risk HPV DNA test is not more sensitive and substantially less specific than repeat cytology [15, 16]. In some studies, HPV-based triage of LSIL in women older than 30–35 years has been suggested [21, 22], but other studies have not confirmed this [23, 24]. Defining the best strategy to triage LSIL lesions is, therefore, identified as a priority for research [25]. In a Norwegian study of 522 women with LSIL, 207 had biopsies and 125 of them had CIN2+. The sensitivity and specificity of repeat cytology (ASC-US or worse) were 85.7% (95% (CI): 72.1, 92.2) and 54.4 % (95% CI: 46.9, 61.9), respectively. The sensitivity and specificity of the HPV mRNA test were 94.2% (95% CI: 88.7, 99.7) and 86.0% (95% CI: 81.5, 90.5), respectively. The PPV of repeated cytology was 38.4% (95% CI: 29.9, 46.9) compared to 67.0% (95% CI: 57.7, 76.4) of the HPV mRNA test. In conclusion, five genotype HPV E6/E7 mRNA testings were more sensitive and specific than repeated cytology in triage of women with LSIL cytology. In addition, the HPV mRNA test showed higher PPV. These data indicate that the HPV mRNA test is a better triage test for women with LSIL than repeated cytology [15, 16]. In a meta-analysis by Arbyn, the pooled sensitivity and specificity of APTIMA to triage LSIL were 96.7% (95% CI: 91.4–98.9%) and 38.7% (95% CI: 30.5–47.6%) for CIN3+. APTIMA was as sensitive as HC2 but more specific (ratio: 1.35; 95% CI: 1.11–1.66). In both triage of ASC-US and LSIL, APTIMA is as sensitive but more specific than HC2 for detecting cervical pre-cancer [14]. In follow-up of patients with negative colposcopy/negative cervical histology, the five genotype HPV E6/E7 mRNA tests have a higher specificity and a higher PPV for CIN2+ than repeated cytology, suggesting a ‘test and treat’ approach for HPV mRNA-testing in women above 40 years [26]. In clinical studies, histology is used as a gold standard for detecting CIN2+. Unfortunately, colposcopy does not have optimal sensitivity for CIN2+. The National Health Service Cervical Screening Programme (NHSCSP) Guidelines for Colposcopy and Programme Management, which guides British practice, asks for evidence of a colposcopic accuracy of 65% [27]. Zuchna, reported 66.2% sensitivity of CIN2+ when up to three guided cervical biopsies were taken regarded as a diagnostic test with the cone specimen as reference standard [28]. Using digitised cervical images from 919 women referred for equivocal or minor cytologic abnormalities into the ASCUS-LSIL Triage Study, Massad, reported 39% sensitivity for CIN2+ [29]. Hence, all women with negative colposcopy and biopsies after abnormal cytology and/or HPV testing have to be followed.

## HPV mRNA testing in the follow-up after conservative treatment for CIN2/CIN3 lesions

Women conservatively treated for CIN2–3 lesions are considered at high risk of developing invasive carcinoma for many years, and therefore, they require continued surveillance. In particular, between 5% and 20% of women treated for CIN2+ will develop recurrent disease within 3 years [30]. For this reason, an adequate follow-up is mandatory. Currently, despite the now perceived low sensitivity, Pap test is widely used in the follow-up of patients treated for intraepithelial neoplasia and European guidelines for cervical screening policy recommend 6-, 12- and 24-month cytology after CIN treatment [31, 32]. Observational studies demonstrated that HPV-DNA testing has a significant higher efficacy compared to cytology in predicting persistent disease or relapse, and hence, the detection of a persistent infection with hrHPV genotypes has the potential to improve patient management. In point of fact hrHPV-DNA testing is significantly more sensitive (90%) compared with follow-up cytology (70%) [33, 34] and currently ASCCP recommends the use of HPV-DNA testing with cytology (co-testing) at 12 and 24 months following treatment for CIN2/3 [35]. Actually, eradication of the clinical lesion does not necessarily mean eradication of all the infected tissue and up 40% of treated women still remains hr-HPV positive due to the fact that hrHPV-DNA testing does not distinguish between a persistent infection from a new, transient one [36, 37]. This is the reason why hrHPV-DNA testing owns a lower specificity than cytology in identifying relapse or persistent disease. Recent prospective studies have documented that rates of residual or recurrent disease in women with persistent hrHPV 16 and/or 18 are higher than in women with other hrHPV types, stressing the importance of type–specific genotyping determination after treatment for CIN2+ [38–40]. As hrHPV-genotyping assays have become increasingly utilised in clinical practice, further research will be needed to determine whether type-specific HPV results can improve the identification of women most likely at higher risk of developing cervical disease after treatment. A recent systematic review highlights that new HPV infections are a potential source for future disease risk after treatment for cervical pre-cancer and cancer [41]. More recently, it has been suggested that the oncogene activity by hr-mRNA transcripts [42] may be a better indicator of women at risk of persistence or relapse of the disease, showing an higher specificity than DNA-based assays, having a better specificity and negative predicting value (NPV) than hrHPV-DNA testing [43], while others underline that HPV E6/E7 mRNA is not useful to detect relapse [2]. In order to assess the performance of different biomolecular tests, alone or in cotesting with cytology, an Italian prospective study is on-going to evaluate the prediction of persistence/recurrence rate in women treated for CIN2+. Preliminary results seem to indicate that cotesting Pap + HPV E6/E7 mRNA shows the best specificity, while hrHPV genotyping retains the higher prediction for CIN2+ persistent or recurrent disease.

## Conclusions

In general, HPV E6/E7 mRNA testing has been demonstrated in several published experiences to have the characteristic of an accurate diagnostic tool in the field of cervical cancer prevention [44]. For this reason, current evidence suggests that mRNA testing may be regarded with promising possibilities in terms of implementation of the diagnostic accuracy of pre-neoplastic cervical lesions in different settings [45–50], including glandular lesions and adenocarcinoma [51]. Primary screening has obviously been investigated more widely, representing the most interesting application option in view of the recent evidence-based shift from cytology to molecular-based screening programmes worldwide. Compared to hrHPV-DNA testing, which actually represents the most validated alternative to cytology in screening settings, mRNA tests present the valuable improvement of a better specificity, and consequently, higher positive predictive value (PPV) towards highgrade cervical lesions (CIN2+) [52]. In terms of test of cure after treatment, though with the significant limitation of a lack of large controlled trials, interesting preliminary data indicate a possible integration of mRNA testing with cytology. HPV E6/E7 mRNA testing may serve as a more specific discriminator between transient cervical dysplasia and potentially progressive lesions. Accordingly, testing for high-risk HPV E6/E7 mRNA might reduce the psychological burden associated with HPV-DNA testing [53].

In conclusion, since the beginning of the molecular era in the prevention of cervical cancer, many steps forward have already been made to overcome the limitations of cervical cytology alone; mRNA testing actually represents a new challenge, with the promising possibility of being integrated in the pool of valuable molecular tools that will hopefully lead to the elimination of invasive cervical cancer in women in the near future.

## Figures and Tables

**Table 1.**
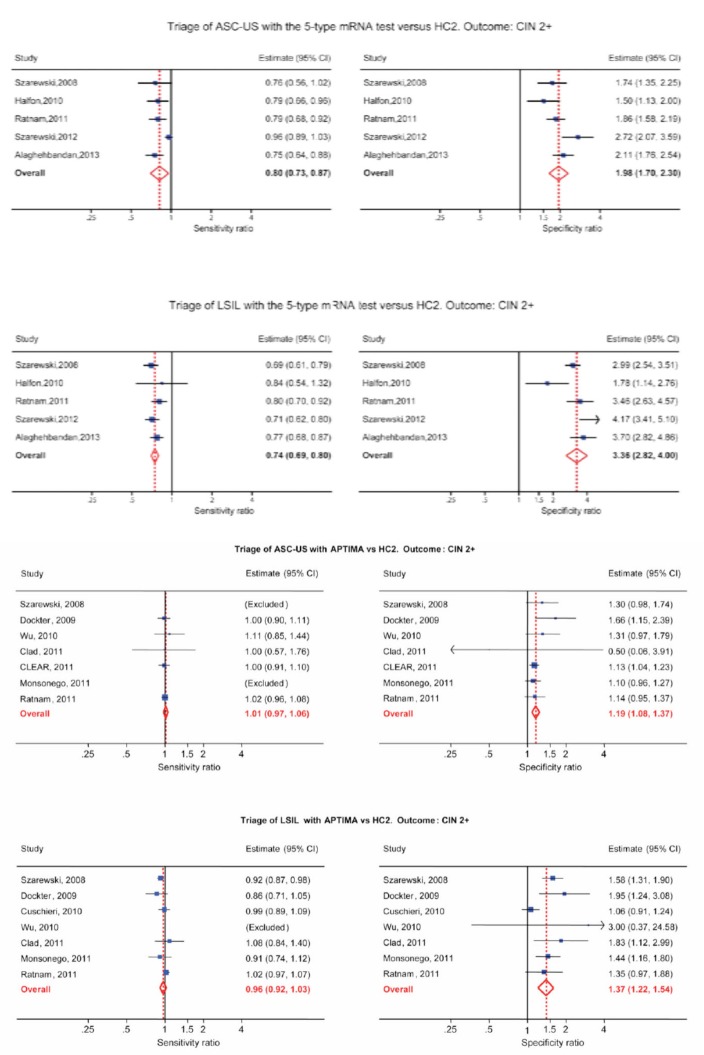
Performance of E6/E7 HPV mRNA and DNA tests.
